# Interaction of Polysialic Acid with CCL21 Regulates the Migratory Capacity of Human Dendritic Cells

**DOI:** 10.1371/journal.pone.0006987

**Published:** 2009-09-14

**Authors:** Marieke Bax, Sandra J. van Vliet, Manja Litjens, Juan J. García-Vallejo, Yvette van Kooyk

**Affiliations:** Department of Molecular Cell Biology and Immunology, VU University Medical Center, Amsterdam, The Netherlands; Institut de Pharmacologie et de Biologie Structurale, France

## Abstract

Dendritic cells (DCs) are the most potent antigen-presenting cells (APCs). Immature DCs (iDCs) are situated in the periphery where they capture pathogen. Subsequently, they migrate as mature DCs (mDCs) to draining lymph nodes to activate T cells. CCR7 and CCL21 contribute to the migratory capacity of the DC, but it is not completely understood what molecular requirements are involved. Here we demonstrate that monocyte-derived DCs dramatically change ST8Sia IV expression during maturation, leading to the generation of polysialic acid (polySia). PolySia expression is highly upregulated after 2 days Toll-like receptor-4 (TLR4) triggering. Surprisingly, only immunogenic and not tolerogenic mDCs upregulated polySia expression. Furthermore, we show that polySia expression on DCs is required for CCL21-directed migration, whereby polySia directly captures CCL21. Corresponding to polySia, the expression level of CCR7 is maximal two days after TLR4 triggering. In contrast, although TLR agonists other than LPS induce upregulation of CCR7, they achieve only a moderate polySia expression. In situ we could detect polySia-expressing APCs in the T cell zone of the lymph node and in the deep dermis. Together our results indicate that prolonged TLR4 engagement is required for the generation of polySia-expressing DCs that facilitate CCL21 capture and subsequent CCL21-directed migration.

## Introduction

The transition of immature DCs (iDCs) to mature DCs (mDCs) is well known to endow dendritic cells (DCs) with the capacity to couple innate to adaptive immune responses. Resting iDCs reside in the periphery, where they sense for pathogen by TLRs [1]. Upon pathogen recognition, a signaling cascade initiates the DC maturation process, characterized by the upregulation of MHC class II and co-stimulatory molecules. In order to initiate the adaptive immune response, DCs travel through the lymphatics to the draining lymph node. In the lymph node, they arrive as fully matured DCs, able to promote the activation of naïve T cells through antigen presentation [2]. Therefore, the phenotypic and functional changes associated with maturation are of critical importance for a proper immune response.

Little is known about posttranslational protein modifications that could contribute to the functional switch of iDCs to mDCs. Several processes, such as T cell activation and differentiation [3;4] as well as DC maturation [5;6] have been reported to be accompanied by programmed remodeling of their cell surface glycosylation. Glycosylation is a highly regulated process that takes place in the Golgi apparatus by the step-wise addition of carbohydrates by glycosyltransferases to maturing glycoproteins and glycolipids [7]. Sialyltransferases comprise a large family of glycosyltransferases that are responsible for the capping of glycans with terminal sialic acids. DC maturation results in dramatic changes in the gene expression profile of sialyltransferases, and amongst them, ST8Sia IV appears to show the largest differences [5]. ST8Sia IV is an α-N-acetylneuraminate α2,8-sialyltransferase that catalyzes the transfer of sialic acid to a sialylated glycan to generate polysialic acid (polySia) [8].

PolySia is a linear homopolymer of α2,8-linked sialic acids, ranging up to 300 residues [9;10]. Although polySia expression was originally thought to be exclusive expressed on NCAM on neuronal cells, it has recently been found on several other glycoproteins, such as the α-subunit of the voltage-sensitive sodium channel in the brain [11], CD36 in human milk [12] and neuropilin-2 on DCs [13]. Polysialylation of neuropilin-2 was shown to negatively regulate the activity and T cell proliferative capacity of DCs [13].

Migration of DCs from the periphery to the lymph node is regulated by the expression of CCL21 in the secondary lymphoid organs and its receptor CCR7expressed by mDCs [14]. Recently, the sialomucin PSGL-1 has been described to interact with CCL21 to facilitate the homing of T cells [15]. Although the molecular mechanism by which PSGL-1 captures CCL21 and contributes to chemotaxis is still unclear, it was suggested that the negative charge contributed by the sulfate groups on PSGL-1 may play a role, in analogy with the capacity of highly sulfated glycosaminoglycans to capture CCL21 [16]. Based on these findings we hypothesized that the upregulated expression of the highly negatively charged polySia induced during maturation could play a role in chemokine capture in order to facilitate DC migration to the lymph node.

In this study we have investigated the kinetics of polySia expression during DC maturation and on several DC subsets. We demonstrate that polySia on O-linked glycans on monocyte-derived DCs is required for CCL21-directed migration through binding of CCL21 to sialic acids on the DC surface. Additionally, polySia expressing APCs were found in human tissue sections of skin and lymph node.

## Results/Discussion

### DCs matured for 2 days with LPS express high levels of polySia on O-glycans

**Figure 1:**
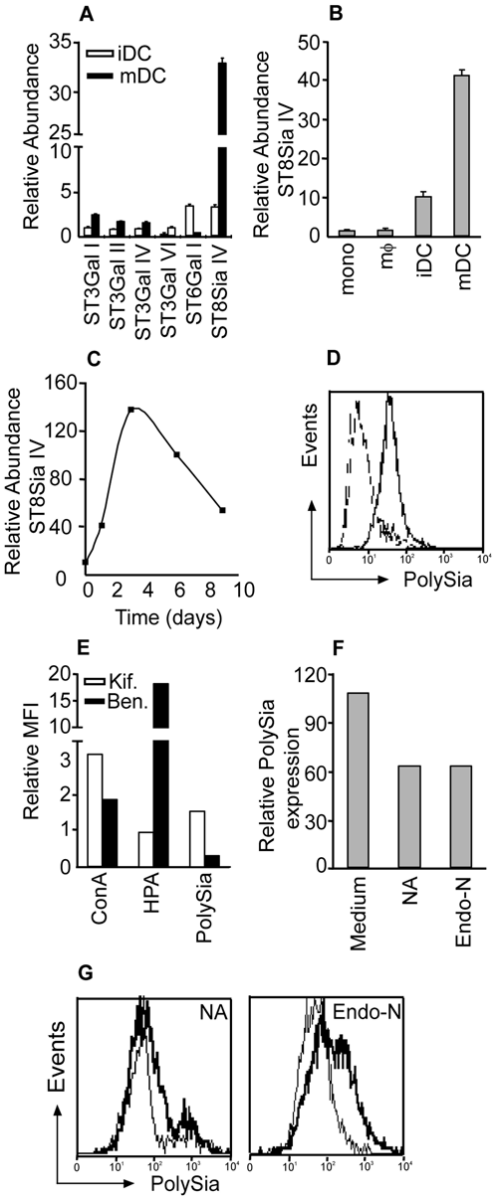
DCs matured for 2 days with LPS express high levels of O-linked polysialic acid. (A) mRNA levels of sialyltransferase genes of iDCs or mDCs triggered for 1 day with LPS were determined by quantitative RT-PCR. Averages of 6 donors are depicted. (B) Expression levels of ST8Sia IV in myeloid cells as measured by quantitative RT-PCR (N = 3). (C) Time course of ST8Sia IV expression after LPS triggering, as analyzed by quantitative RT-PCR. One representative donor is shown. (D) Expression levels of polySia by iDCs (dotted line) and DCs matured for 2 days with LPS (bold line) or 1 day with LPS (thin line), as measured by flow cytometry with moAb 735. Results are representative of 5 independent experiments. (E) Flow cytometric analysis of the glycosylation of mDCs treated with kifunensine to block N-glycan synthesis, or with benzyl-α-GalNAc to block O-glycan synthesis using the lectins Con-A (high-mannose structures) or HPA (Tn antigen). Depicted are the relative fluorescence values of kifunensine or benzyl-α-GalNAc treated cells compared to untreated cells. Relative fold increase in expression of polySia after maturation (MFI mDC/MFI iDC) was analysed with mAb 735 (polySia). One representative donor is shown. (F, G) 50% of PolySia expressed by mDCs is removed with neuraminidase and Endo-neuraminidase treatment as analyzed by flow cytometry with moAb 735. One out of 3 independent experiments is shown.

DC maturation is associated with a functional change from antigen capture towards migration to the lymph node and activation of T cells. We recently observed that maturation also results in a dramatic reprogramming of the glycosylation machinery, especially with regard to sialylation [5]. DC maturation after triggering of TLR4 with LPS resulted in the upregulation of α2,3- and α2,8-linked sialyltransferase transcripts, whereas ST6Gal I transcripts, encoding for an α2,6-sialyltransferase, were down regulated, as measured by quantitative RT-PCR (Figure 1A). Interestingly, ST8Sia IV transcripts levels were much higher than the remaining sialyltransferase transcripts. ST8Sia IV is required for the biosynthesis of the unusual glycan structure polySia [8], [17]. In contrast to monocytes, macrophages and iDC, the high expression levels of ST8Sia IV transcripts appeared to be exclusive for mDC (Figure 1B). In order to determine the kinetics of ST8Sia IV upregulation during LPS-induced DC maturation, quantitative RT-PCR analysis of ST8Sia IV mRNA was performed at different time points after LPS treatment. Already 1 day after LPS treatment ST8Sia IV transcripts were upregulated, which are further enhanced on day 2, whereas the strongest upregulation was observed 3 days after LPS treatment (Figure 1C). In contrast to ST8Sia IV mRNA expression, no polySia expression was found on the cell membrane of iDC and mDC matured for 1 day with LPS. Cleary polySia levels were enhanced on DC matured with LPS for 2 days as detected by staining with the specific antibody 735 [18] (Figure 1D), indicating that at least one day is necessary for the appropriate glycoprotein turnover and to allow newly synthesized polySia containing glycoproteins to reach the extracellular membrane. Since the viability of *in vitro* DC might be compromised after a prolonged exposure to LPS, we have chosen to investigate the effects of high levels of polySia on DC only after 2 days of LPS exposure.

PolySia is commonly found on N-linked core structures attached to NCAM on neuronal cells [9], but it has also been described to be attached to O-linked glycans on CD36 [12]. To determine whether polySia on mDCs is attached to O- or N-linked glycans, we used the glycosylation inhibitors kifunensine and benzyl-α-GalNAc. The Golgi mannosidase-inhibitor kifunensine [19] inhibits the maturation of N-glycans rendering structures consisting of high-mannose. O-glycan synthesis was blocked by treating the cells with benzyl-α-GalNAc [20], an analogue of GalNAc that can not be used as substrate by subsequent glycosyltransferases. Efficiency of interference with glycosylation was confirmed using the high-mannose-specific lectin Con A and the GalNAc-specific lectin HPA. Highly increased polySia expression levels were observed on kifunensine (N-linked glycosylation) treated mDCs, while benzyl-α-GalNAc (O-linked glycosylation) efficiently blocked the maturation-induced polySia upregulation (Figure 1E). The apparently contradictory increase in polySia expression induced by kifunensine could be explained by a better availability of substrates for O-linked glycan synthesis upon N-glycan biosynthesis blockade. Together, these data indicate that polySia on mDC is O-linked to glycoproteins. Treatment of mDCs with neuraminidase (NA), removing α2,3-, α 2,6-, and α2,8-linked sialic acid, or Endo-neuraminidase (Endo-N) specific for polySia, resulted in 50% lower polySia expression, demonstrating the specificity of the moAb 735 (Figure 1F and 1G).

### CCL21 binding to sialic acids is required for CCL21-directed migration of mDCs

**Figure 2:**
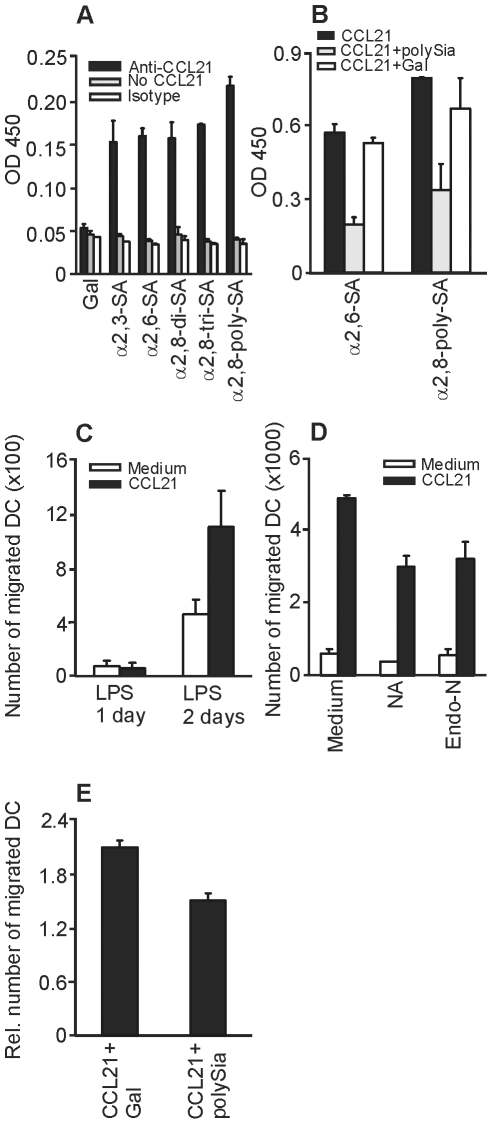
CCL21 binding to sialic acid is required for CCL21-directed migration of DCs. (A) CCL21 binding to coated PAA-conjugated glycans was analyzed by anti-CCL21 antibody and peroxidated goat anti-mouse antibody binding. As a control, CCL21 was substituted by PBS (grey bar) or a murine IgG1 isotype control (white bar) (N = 4). (B) Binding of CCL21 pre-incubated with PBS (black bar), PAA-conjugated polySia (grey bar) or PAA-conjugated galactose (white bar) to coated PAA-conjugated α2,6-linked sialic acid or α2,8-linked polySia. CCL21 binding was analyzed by anti-CCL21 antibody and peroxidated goat anti-mouse (N = 2). (C) DC migration to CCL21 was measured in a transwell assay. Depicted are the numbers of migrated DCs matured for 1 day with LPS (not expressing polySia) or for 2 days with LPS (expressing polySia) to medium (white bar) or medium supplemented with CCL21 (black bar) (N = 4). (D) Migration of mDCs (2 days LPS) treated with NA or Endo-N to medium (white bar) or medium supplemented with CCL21 (black bar) (N = 4). (E) Migration of mDCs (2 days LPS) to CCL21 in the presence of soluble galactose or α2,8-linked polySia (N = 4). Depicted is the relative migration of mDCs towards CCL21 compared to medium controls.

It has been reported that some cytokines have carbohydrate-binding properties. These cytokine-glycan interactions often involve sialic acids, as demonstrated by the binding of IL-1α to disialylated di-antennary N-glycans and IL-7 to a mucin rich in sialyl-Tn antigen [21]. The chemokine CCL21 shows binding to glycosaminoglycans [22] and recently it has been demonstrated that CCL21 binds the glycoprotein PSGL-1 on T cells to facilitate the migration of T cells to secondary lymphoid organs [15]. In order to investigate if CCL21 directly binds to sialic acids and in particular to polySia, we analysed CCL21 binding to sialylated glycan-conjugated polyacrylamide probes (α2,8-di-, tri-, and α2,8-polysialylated). As a control, polyacrylamide probes carrying the neutral monosaccharide galactose were used. CCL21 did not bind galactose, whereas the binding of CCL21 to sialic acid was clearly present and positively correlated to the amount of sialic acids present on the glycan structure (Figure 2A). The binding of CCL21 to coated α2,6-linked sialic acid or α2,8-linked polySia could be inhibited by the addition of soluble α2,8-linked polySia, whereas the addition of soluble galactose did not affect CCL21 binding to sialic acids (Figure 2B).

Since DC migration is directed by CCL21 binding to CCR7 on DCs and we observed direct binding of CCL21 to sialic acids, we hypothesized that polySia on mDCs might promote DC migration towards CCL21. To investigate this, the spontaneous and the CCL21 directed migratory response of DCs with or without polySia expression was measured in a transwell chemotaxis assay. The migration of mDCs triggered for 1 day with LPS, (expressing low polySia levels) were compared to the migration of mDCs treated for 2 days with LPS (expressing high polySia levels). The migratory response of mDCs treated for 2 days with LPS was clearly higher than that of the mDCs with low polySia content (Figure 2C). To demonstrate polySia requirement for CCL21-induced DC migration, mDCs triggered for 2 days with LPS were treated with NA or Endo-N to remove sialic acids. The reduction in CCL21-induced migration (Figure 2D) paralleled the reduction in polySia expresssion achieved by these treatments (Figure 1F), demonstrating the involvement of these structures in the capture of CCL21 to facilitate migration. To exclude that NA or Endo-N treatment interferes with CCR7 function, we added soluble galactose or polySia to CCL21-supplemented medium. The migration of mDC triggered for 2 days with LPS to CCL21-supplemented medium was inhibited with soluble polySia compared to the addition of soluble galactose (Figure 2E). In conclusion, we demonstrate that CCL21 directly binds to sialic acids, probably facilitated by the negative charge of sialic acids. PolySia expression on mDCs could thus create a negatively charged capture mechanism to efficiently promote CCL21-directed migration.

### PolySia is primarily upregulated on DCs matured for 2 days with TLR4 ligands and not present on tolerogenic DCs

**Figure 3:**
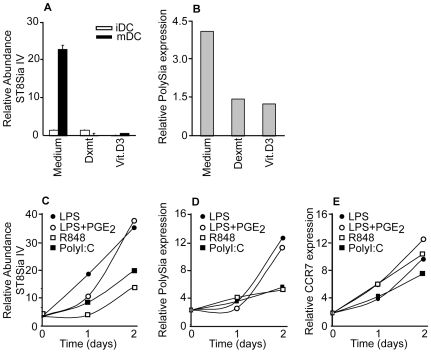
Polysialic acid and CCR7 expression levels on different DC subsets. (A) Expression levels of ST8Sia IV (N = 3) and (B) polySia expression of immunogenic mDCs and dexamethasone or vitamin D3 tolerogenic mDCs, as measured by RT-PCR and flow cytometry respectively. One representative donor out of three is shown. (C) ST8Sia IV expression levels in time of DCs matured with different TLR ligands as measured by RT-PCR. (D) PolySia expression levels in time on DCs matured with different TLR ligands as measured by flow cytometry with mAb 735. One representative donor is shown. (E) CCR7 expression levels in time on DCs matured with different TLR ligands were measured by flow cytometry. One representative donor is shown. For all experiments the relative antibody binding compared to the secondary antibody control is depicted.

*In vivo*, different functional DC phenotypes can be distinguished. Tolerogenic DCs maintain tolerance by deleting self-reactive T cells and generating regulatory T cells, whereas immunogenic DCs induce immunity by activating effector and memory T cells [23]. The migratory properties of DC subsets also vary, as pretreatment of DCs with tolerogenic stimuli results in a reduced capacity to migrate upon exposure to LPS and a loss of the ability to reach draining lymph nodes [24]. In previous experiments we clearly observed enhanced polySia expression on DCs triggered for 2 days with LPS. To establish the conditions that allow the highest polySia upregulation, we investigated next to immunogenic DC the potential of tolerogenic DCs to express polySia. The expression levels of ST8Sia IV, as well as the cell surface expression of polySia expression, was evaluated on different *in vitro* cultured DCs. DCs matured with LPS for 2 days were used as a model for immunogenic DCs and as a model for tolerogenic DCs, DCs generated in the presence of dexamethasone or 1,25(dihydroxi-) vitamin D3 [25;26] were used. Surprisingly, only immunogenic DCs but not tolerogenic DCs expressed high levels of ST8Sia IV transcripts (Figure 3A). Similar to the expression of ST8Sia IV, polySia is only expressed on the cell surface of immunogenic and not on tolerogenic DCs (Figure 3B).

Next, we studied whether other TLR antagonists similarly enhance polySia expression as the TLR4 ligand LPS. DCs were treated with MyD88 dependent-TLR7/8 ligand R848 as well as MyD88-independent/TRIF-dependent TLR3 ligand Poly I∶C [27]. We also investigated polySia expression of DCs matured with LPS in combination with PGE_2_, since the presence of PGE_2_ during DC maturation has been described to control the migratory capacity [28]. Although LPS induced the strongest upregulation of ST8Sia IV (Figure 3C) and polySia expression on the DC surface (Figure 3D), Poly I∶C and R848 also enhanced expression although clearly to a lower extent. The combination of PGE_2_ and LPS did not further increase the LPS-mediated upregulation of ST8Sia IV and polySia expression, indicating that LPS is the crucial factor associated with polySia upregulation on DCs.

The receptor of CCL21 on DCs is CCR7 [14]. CCR7 is induced upon DC maturation whereas the expression of other chemokine receptors is downregulated during DC maturation [29;30]. When we analysed the regulation of CCR7 expression, we detected similar kinetics to that of polySia, showing the highest expression after 2 days maturation with LPS. However, in contrast to the induction of polySia expression, all TLR-ligands tested promoted equally high CCR7 expression levels (Figure 3E). Our data indicate that TLR4 agonists provide the requirements for optimal polySia and CCR7 expression on DCs that allow efficient CCL21-induced migration of DCs. Furthermore, our data demonstrate that TLR4 maturation of tolerogenic DCs upregulates CCR7 but not polySia, indicating that probably tolerogenic DCs are less capable of capturing CCL21 and may thus need higher concentration gradients of CCL21 in order to perform CCR7-mediated migration.

### APCs expressing polySia are present in human tissue

**Figure 4:**
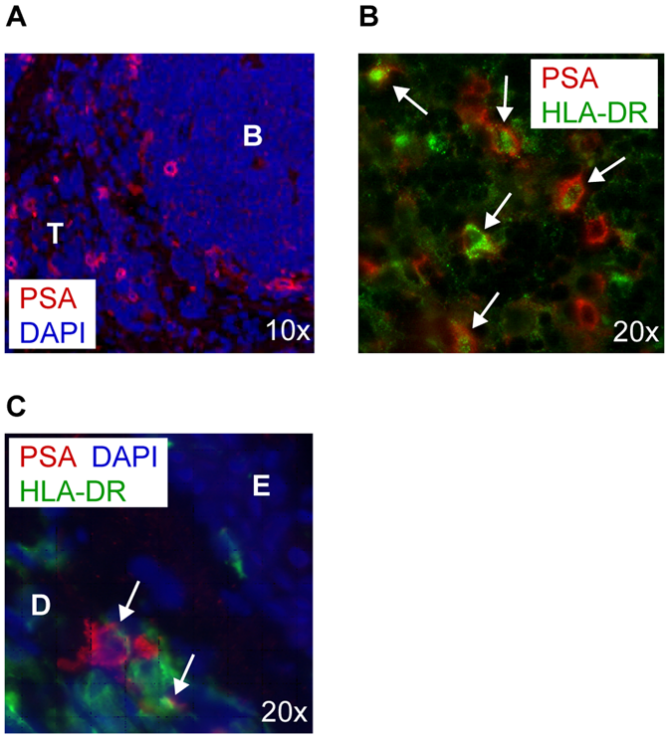
PolySia^+^ antigen presenting cells are present in human tissue. (A) Tissue biopsies of human lymph node were stained with moAb 735 (red). PolySia positive cells in the lymph node are located in the T cell area (T) but not in the B cell follicle (B). Nuclei are visualized in blue (10×). (B) Human lymph node was double stained using moAb 735 (red) and anti-HLA-DR (green). Arrows indicate double positive cells. Nuclei are visualized in blue (20×). (C) Tissue biopsies of human skin were double stained with moAb 735 (red) and anti-HLA-DR (green). Only deep in the dermis (D) and not in the epidermis (E) polySia^+^ cells can be detected. Arrows indicate double positive cells. Nuclei are visualized in blue (20×).

To investigate if polySia-expressing cells are present in human tissue, lymph node and skin biopsies were stained with anti-polySia and anti-HLA-DR monoclonal antibodies. In the lymph node, polySia-expressing cells could be identified in the T cell zone but not in the B cell area (Figure 4A). These polySia-expressing cells in the T cell zone of the lymph node were APCs, expressing HLA-DR (Figure 4B). In skin, polySia-expressing APCs were located only in the dermis (Figure 4C). Potentially, the polySia-expressing APCs in the deep dermis may traffic during inflammation in a CCL21-directed manner to the lymph node, where also polySia-expressing HLA-DR^+^ cells could be found, followed by presentation of their antigen to T cells [14].

Based on the results of this study, the expression of polySia as a marker for optimal migratory mDCs should be considered. Especially in vaccination based therapies in which efficient migration of *ex vivo* generated DCs is required, it should be taken into account to analyse next to CCR7 the polySia expression levels on the DCs. In conclusion, we demonstrate that the expression of polySia on the cell surface of fully mature DCs following TLR4 triggering for 2 days captures CCL21 to optimally direct DC migration towards the lymph nodes.

## Material and Methods

### Ethics statement

The study was approved by the VU university medical center (VUmc) Amsterdam and the Commissie Wetenschappelijk Onderzoek (CWO) by written consent. A written informed consent was obtained from the healthy donors for the use of samples.

### Dendritic cell culture

Monocytes were isolated from healthy donors (Sanquin) through Ficoll gradient centrifugation and positive selection of CD14^+^ cells using MACS sorting (Miltenyi Biotec). Isolated monocytes were cultured in RPMI 1640 (PAA laboratories) containing 10% FCS (BioWhittaker), IL-4 (500 U/ml; Biosource) and GM-CSF (800 U/ml; Biosource) for 7 days. Tolerogenic DC were generated in the presence of dexamathasone (10^−6^ M, Sigma-Aldrich) or calcitriol (1,25(OH)_2_,D_3_, 10^−8^ M, Sigma-Aldrich). At day 5 or day 6 maturation was induced by the addition of 10 ng/ml LPS (*E. Coli*; Sigma-Aldrich) for 24 or 48 hours.

### Sialidase treatment

Endo-N (AbCys) was used to specifically cleave off polySia. Cells were pre-treated with the enzyme (0.7 U/ml) in medium overnight and again 4 hours prior to the experiment. In order to cleave off α2,3-, α2,6- and α2,8-linked sialic acids, cells were pre-incubated with the *Vibrio cholera* neuraminidase (Roche, 2.5×10^−2^ U/ml) for 1 hour at 37°C.

### Real-Time PCR

Cells were lysed and mRNA was isolated using an mRNA Capture kit (Roche). cDNA was synthesized using the Reverse Transcription System kit (Promega) following manufacture's guidelines. Oligonucleotides were designed using the Primer Express 2.0 software (Applied Biosystems) and synthesized by Invitrogen Life Technologies (Invitrogen). Real-Time PCR analysis was performed as previously described with the SYBR Green method in an ABI 7900HT sequence detection system (Applied Biosystems), using GAPDH as endogenous reference.

### Flow cytometry

DCs (5×10^4^/well) were incubated with 10 µg/ml antibody 735 (kindly provided by Dr. Mühlenhoff) or anti-CCR7 (BD) for 45 min. at RT in PBS with 1% BSA (Fluka Biochemika). Cells were counter stained with a secondary FITC-labeled goat anti-mouse IgG (Zymed) for 30 min. at RT in PBS with 1% BSA and analyzed by flow cytometry (FACS Calibur, BD). Lectin stainings with ConA (*Concanavalin A*, Sigma-Aldrich) and HPA (*Helix pomatia agglutinin*, Sigma-Aldrich) were performed in TSM (20 mM Tris (pH 7.4), 150 mM NaCl, 1 mM CaCl_2_, and 2 mM MgCl_2_). Alexa 488-labeled streptavidin was used to detect lectin binding.

### O- and N-linked glycan modification

DC cultures were supplemented with 2 µg/ml of the mannosidase I inhibitor Kifunensine (*Kitasatosporia kifunensine*; Calbiochem) for at least 5 days to induce high mannose type glycosylation of N-linked glycoproteins. O-glycosylation was abrogated by the addition of 2 mM/ml Benzyl-α-GalNac (Sigma-Aldrich) for at least 5 days.

### Binding assay

Nunc maxisorp 96-wells ELISA plates (Nalge Nunc International) were coated overnight at 4°C with 10 µg/ml galactose, α2,3-linked sialic acid, α2,6-linked sialic acid or α2,8-linked di-, tri- or polySia coupled to biotinylated Polyacrylamide (Lectinity). Wells were blocked for 30 min. at 37°C with TSM (20 mM Tris-HCl, 150 mM NaCl, 1 mM CaCl_2_, 1 mM MgCl_2_) containing 1% BSA (Fluka Biochemika). After washing 0.1 µg/ml CCL21 (Peprotech) in TSM with 1% BSA was added for 2 hours at RT. For blocking studies, 0.1 µg/ml CCL21 was pre-incubated with 25 µg/ml galactose or α2,8-linked polysialic acid coupled to biotinylated Polyacrylamide (Lectinity) for 45 min. at 37°C in TSM. CCL21 binding was determined by incubating 5 µg/ml anti-CCL21 (R&D) in TSM with 1% BSA for 2 hours at RT, followed by a detection step containing 10 µg/ml peroxidated goat-anti-mouse antibody. The reaction was developed and optical density was measured at 450 nm.

### Chemotaxis assay

2×10^5^ cells in 100 µl RPMI 1640 (PAA laboratories) containing 10% FCS (BioWhittaker) were added to the upper well of a Transwell (8.0 µm pores, Costar). Transwells were placed in a 24-well plate with 600 µl full medium containing 100 ng/ml CCL21 (PeproTech) and incubated for 4 hours at 37°C in 5% CO_2_. Migrated DC were collected from the bottom well and counted using flow cytometry (BD). For blocking studies, both the lower well containing CCL21 (100 ng/ml) and the upper well containing the DCs were pre-treated with 10 µg/ml galactose or α2,8-linked polysialic acid coupled to Polyacrylamide (Lectinity) for 45 min. at 37°C before performing the migration assay.

### Immunohistochemistry

Human tissue sections (7 µm) were fixed in acetone and blocked with goat serum prior to staining. Antibody 735 or rat anti-human HLA-DR (Santa Cruz Biotechnology) were added at 10 µg/ml in PBS containing 1% BSA (Fluka Biochemika) for 60 min. at 37°C. Sections were counter stained using secondary isotype specific-Alexa antibodies for 30 min. at room temperature, and analysed by fluorescence microscopy (Leica microsystems).
